# Unmasking a hidden culprit: late-presenting congenital diaphragmatic hernia beyond infancy: A case report and literature review

**DOI:** 10.1097/MD.0000000000037450

**Published:** 2024-03-22

**Authors:** Wen-Harn Koh, Po-Jui Ko, Yu-Tsun Su, Yu-Cheng Tsai, Ho-Poh Kek, Ching-Chung Tsai

**Affiliations:** a Department of Pediatrics, E-Da Hospital, I-Shou University, Kaohsiung City 82445, Taiwan; b Division of Pediatric Surgery, Department of Surgery, E-Da Hospital, I-Shou University, Kaohsiung City 82445, Taiwan; c School of Medicine for International Students, I-Shou University, Kaohsiung City 82445, Taiwan.

**Keywords:** late-presenting congenital diaphragmatic hernia, tension gastrothorax, tension pneumothorax

## Abstract

**Background:**

Congenital diaphragmatic hernia (CDH) is a rare congenital anomaly with abnormal diaphragm development, typically diagnosed prenatally or soon after birth. Late-presenting CDH presents diagnostic challenges due to nonspecific symptoms that can lead to misdiagnoses.

**Methods:**

This report discusses a 35-month-old female initially presenting with predominant gastrointestinal symptoms and minimal respiratory distress. Initial radiographic findings suggested a left tension pneumothorax, prompting further investigation.

**Results:**

Subsequent diagnostic efforts revealed a Bochdalek-type left CDH, with several abdominal organs herniated into the thoracic cavity. The case was managed through laparotomy, where herniated contents were successfully repositioned into the abdominal cavity. This intervention underscores the need for high clinical suspicion and the importance of distinguishing between similar presentations, such as tension pneumothorax and tension gastrothorax, which require different management strategies.

**Conclusion:**

The case illustrates the importance of considering CDH in differential diagnoses for older pediatric patients with atypical symptoms. Early recognition and appropriate management are key to improving patient outcomes.

## 1. Introduction

Congenital diaphragmatic hernia (CDH) is a rare congenital anomaly characterized by a defect in the diaphragm, allowing abdominal organs to migrate into the thoracic cavity. This results in the compression of lung tissue, leading to pulmonary hypoplasia and the development of pulmonary hypertension. In addition to its respiratory implications, CDH may manifest with associated anomalies in the cardiovascular, neurological, and musculoskeletal systems.^[1]^ The primary subtypes of CDH include posterolateral (Bochdalek), anterior (Morgagni), and central. Although over 60% of CDH cases can be diagnosed prenatally or shortly after birth,^[2]^ the identification of late-presenting CDH poses a clinical challenge, primarily due to its ambiguous symptomatology. Symptoms such as cyanosis, increased respiratory rate, and elevated heart rate may obscure a timely diagnosis. This case report aims to highlight the diagnostic complexities associated with late-presenting CDH, underscoring the importance of considering it in the differential diagnosis, particularly when gastrointestinal symptoms are prominent initially.

## 2. Case report

**Figure 1.**
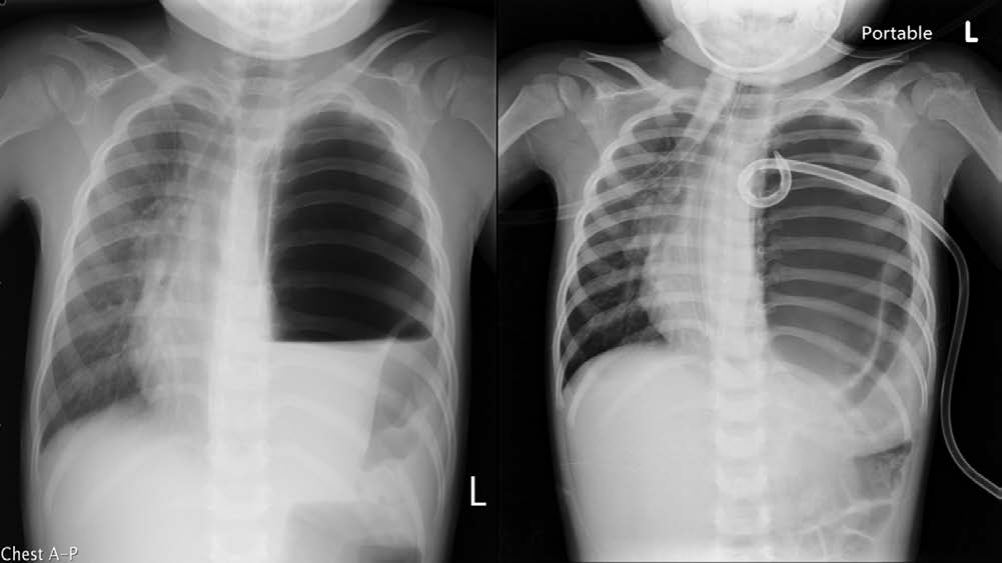
The left panel displays a radiograph showing an absence of lung markings and the presence of an air-fluid level in the left lung. The right panel depicts an unsuccessful attempt at decompressing the left lung using pigtail catheter insertion.

A 35-month-old female was admitted to the emergency department presenting with nausea and episodic abdominal pain persisting for roughly half a day. Her medical history was unremarkable with no prior serious respiratory or gastrointestinal complications. She produced stools that were yellowish, foul-smelling, and mucoid, but without any traces of blood. Clinical assessments on admission revealed a body temperature of 35.7°C, heart rate of around 120 beats/minute, a blood pressure reading of 108/80 mm Hg, and a respiratory rate of 16 breaths/minute. A physical examination indicated an absence of respiratory sounds over the left lung field. Laboratory findings indicated leukocytosis with a white blood cell count at 22.8 × 10^9^/L and neutrophils constituting 74.2%. Additionally, thrombocytosis was observed with platelets at 451 × 10^3^/μL. Elevated aspartate aminotransferase levels were noted at 50 U/L. Chest and abdominal radiographs displayed a clear absence of lung delineations on the left side, accompanied by air-fluid levels. Additionally, there was an evident deviation of the tracheobronchial tree towards the right. A distinct rightward shift in both the esophagus and the heart’s outline was also noted. Based on these findings, a left tension pneumothorax was preliminarily suspected, prompting the placement of a pigtail catheter. However, subsequent chest imaging revealed an incomplete decompression of the left lung, leading to heightened suspicions of a diaphragmatic hernia (Fig. 1).

**Figure 2.**
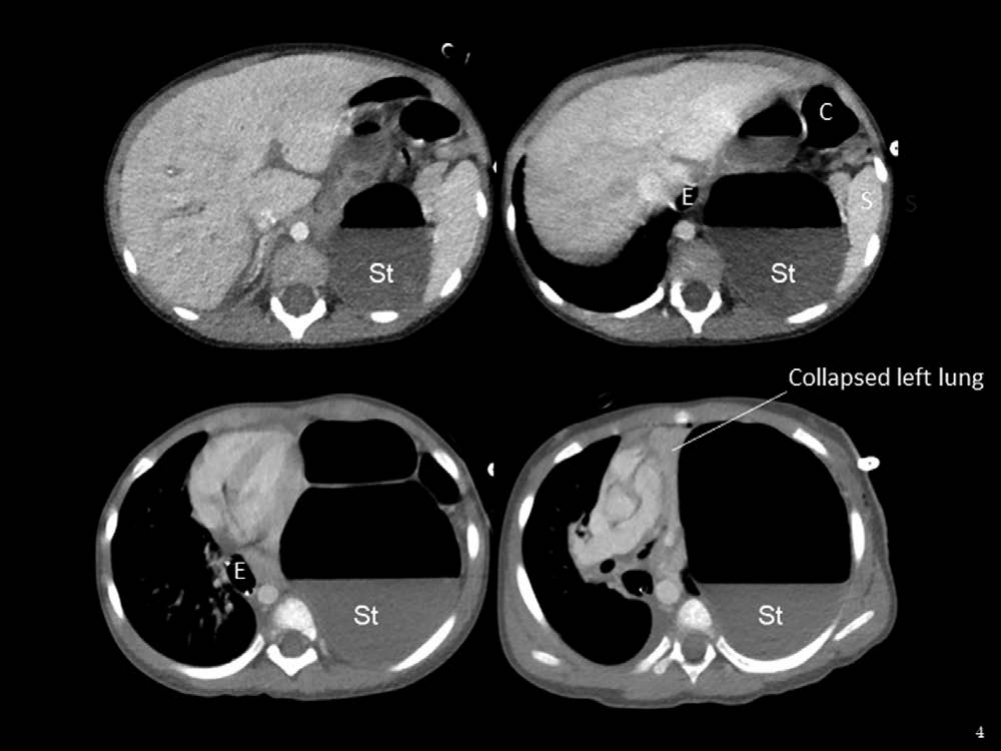
This axial computed tomography scan image with contrast demonstrates a diaphragmatic hernia. Key structures are labeled: E for esophagus, S for spleen, St for stomach and C for colon.

Immediate chest computed tomography scans unequivocally confirmed a CDH, with evident intrusion of the stomach, colon, and spleen into the left thoracic cavity. This herniation was associated with notable atelectasis and hypoplasia of the left lung, as demonstrated by a shrunken left pulmonary artery, a constricted left main bronchus, and a shift of the mediastinum to the right (Fig. 2).

During the laparotomy, a Bochdalek-type left CDH was identified, presenting a defect of 5 × 4 cm, which accounted for roughly 30% of the diaphragmatic surface area. Several organs, including the stomach, colon, omentum, small intestine, and spleen, were found to have migrated into the left thoracic region. postsurgical chest images showcased the full reinflation of the left lung. The patient’s recovery proceeded without complications. Follow-up echocardiography confirmed a structurally and functionally sound heart.

## 3. Discussion

CDH occurs in approximately 2.3 cases per 10,000 live births, underscoring its infrequency while highlighting its clinical importance.^[3]^ The clinical presentation of CDH exhibits notable variations contingent upon its temporal occurrence. Early-presenting CDH is characterized by acute, discernible symptoms, facilitating a relatively straightforward diagnosis. Conversely, late-presenting CDH may manifest as subtle, intermittent, or chronic symptoms, often leading to misattribution to more commonplace pediatric conditions.^[4]^

A comprehensive study by Yoshihiro et al elucidated intriguing patterns in the symptomatology of late-presenting CDH. Among 79 patients with late-presenting CDH, both respiratory and gastrointestinal symptoms were evident, with a discernible correlation between the side of herniation and the predominant symptomatology.^[5]^ Gastrointestinal issues were more prevalent in left-sided hernias, attributed to the displacement of the stomach and intestines, while right-sided hernias were more associated with respiratory symptoms.^[6]^

In diagnosing late-presenting CDH, standard initial evaluations typically involve chest or abdominal radiographs. However, potential misinterpretations may arise, particularly when clinicians do not specifically consider late-presenting CDH in their differential diagnoses. Conditions such as pleural effusion, pneumonia, or pneumothorax may be erroneously considered in lieu of CDH.^[7]^ Distinguishing between pneumothorax and CDH can be particularly challenging, necessitating a careful differentiation between tension pneumothorax and tension gastrothorax due to their distinct treatments and implications.

Clinically, tension pneumothorax manifests as acute respiratory distress, diminished breath sounds, tracheal deviation, distended neck veins, and hypotension. In contrast, tension gastrothorax may exhibit similar respiratory symptoms but often includes abdominal discomfort. Physical examination reveals hyperresonance in tension pneumothorax upon percussion, whereas tension gastrothorax produces a tympanic sound akin to percussing the stomach.^[8]^ Radiographic distinctions between tension pneumothorax and tension gastrothorax were outlined by Anekar et al, highlighting the latter’s characteristic large air pocket compressing the lung, indistinct or elevated hemidiaphragm, and absence of the gastric bubble from the abdominal view.^[9]^

In light of potential diagnostic challenges, a systematic approach to suspected late-presenting CDH is essential. In addition to radiography, the insertion of a nasogastric tube, revealing its tip above the diaphragm when coiled in the herniated stomach, serves as a simple yet effective diagnostic adjunct.^[9,10]^ Gastrointestinal contrast studies further aid in pinpointing the hernia’s location and extent, particularly when concerns exist regarding bowel involvement.^[11]^

Gastrothorax is uncommon in children, with only 16 cases precisely reported in the literature in the PubMed database (Table 1). The majority of reported pediatric cases were male (13 out of 16, accounting for 81%), while only 3 cases involved female patients (3 out of 16, comprising 19%). Nearly all patients (15 out of 16, or 94%) presented with symptoms of respiratory distress, with chest pain and vomiting being the subsequent prevalent manifestations. Notably, 2 cases involved chest tube insertion before a definitive diagnosis, akin to the circumstances in our case. It is noteworthy that 10 out of 16 cases had nasogastric tube insertion before the operation, either for aiding in diagnosis or alleviating gastric pressure.

**Table 1:** A literature review summarizing reported cases of gastrothorax in pediatric patients.

Age (month)	Gender	Symptoms/signs	Chest tube placement before surgery	NG tube placement before surgery	Author
8	Boy	Respiratory distress	**+**		Zedan et al^[12]^
108	Boy	Respiratory distress, chest pain		**+**	Næss et al^[13]^
9	Boy	Respiratory distress, irritable high-pitched cry			Pelech et al^[14]^
24	Boy	respiratory distress, abdominal pain			Song et al^[15]^
96	Boy	respiratory distress, chest pain, nonbilious vomiting			Anekar et al^[9]^
10	Boy	Respiratory distress	**+**		García et al^[16]^
144	Boy	Respiratory distress			Fuller et al^[17]^
18	Girl	Abdominal pain		**+**	Hooker et al^[18]^
53	Boy	Respiratory distress		**+**	Guo et al^[19]^
72	Boy	Respiratory distress		**+**	Guo et al^[19]^
28	Boy	Respiratory distress		**+**	Guo et al^[19]^
156	Girl	Respiratory distress, chest pain, vomiting		**+**	Rathinam et al^[20]^
120	Boy	Respiratory distress, epigastric pain, vomiting		**+**	Ahn et al^[21]^
9	Boy	Respiratory distress		**+**	Sridhar et al^[22]^
29	Boy	Respiratory distress		**+**	Snyder et al^[23]^
2	Girl	Respiratory distress, excessive crying, irritability		**+**	Al Ghafri et al^[24]^
35	Girl	Abdominal pain	**+**		This article

NG tube = Nasogastric tube

## 4. Conclusions

Late-presenting CDH poses a diagnostic challenge owing to its nonspecific symptoms. It is imperative for healthcare professionals to uphold a heightened level of suspicion, especially in older children who present with prominent gastrointestinal symptoms, as observed in this case, and comparatively fewer respiratory symptoms. This vigilance is essential to facilitate timely intervention and enhance overall patient outcomes.

## Author contributions

**Conceptualization:** Wen-Harn Koh, Ching-Chung Tsai.

**Data curation:** Po-Jui Ko, Yu-Tsun Su, Yu-Cheng Tsai, Ho-Poh Kek.

**Investigation:** Po-Jui Ko, Yu-Tsun Su, Yu-Cheng Tsai.

**Methodology:** Po-Jui Ko.

**Project administration:** Wen-Harn Koh.

**Supervision:** Ching-Chung Tsai.

**Validation:** Ching-Chung Tsai.

**Writing—original draft:** Wen-Harn Koh.

**Writing—review & editing:** Ching-Chung Tsai.

## References

[1] Chatterjee D Ing RJ Gien J. Update on congenital diaphragmatic hernia. Anesth Analg. 2020;131:808–21. 31335403 10.1213/ANE.0000000000004324

[2] Cordier AG Russo FM Deprest J. Prenatal diagnosis, imaging, and prognosis in congenital diaphragmatic hernia. Semin Perinatol. 2020;44:51163. 31439324 10.1053/j.semperi.2019.07.002

[3] Paoletti M Raffler G Gaffi MS. Prevalence and risk factors for congenital diaphragmatic hernia: a global view. J Pediatr Surg. 2020;55:2297–307. 32690291 10.1016/j.jpedsurg.2020.06.022

[4] Chang SW Lee HC Yeung CY. A twenty-year review of early and late-presenting congenital Bochdalek diaphragmatic hernia: are they different clinical spectra? Pediatr Neonatol. 2010;51:26–30. 20225535 10.1016/S1875-9572(10)60006-X

[5] Kitano Y Lally KP Lally PA. Congenital Diaphragmatic Hernia Study Group. Manifestations of congenital diaphragmatic hernia appearing later in life. J Pediatr Surg. 2005;40:1839–43. 16338301 10.1016/j.jpedsurg.2005.08.023

[6] Kitano Y Lally KP Lally PA. Congenital Diaphragmatic Hernia Study Group. Late-presenting congenital diaphragmatic hernia. J Pediatr Surg. 2005;40:1839–43. 16338301 10.1016/j.jpedsurg.2005.08.023

[7] Kim DJ Chung JH. Late-presenting congenital diaphragmatic hernia in children: the experience of single institution in Korea. Yonsei Med J. 2013;54:1143–8. 23918563 10.3349/ymj.2013.54.5.1143 PMC3743178

[8] Singh SP Sukesan S Kiran U. Gastrothorax or tension pneumothorax: a diagnostic dilemma. J Emerg Trauma Shock. 2011;4:128–9. 21633581 10.4103/0974-2700.76821 PMC3097562

[9] Anekar AA Nanjundachar S Desai D. Case report: late-presenting congenital diaphragmatic hernia with tension gastrothorax. Front Pediatr. 2021;9:618596. 33937144 10.3389/fped.2021.618596 PMC8081028

[10] Berman L Stringer D Ein SH. The late presenting paediatric Bochdalek hernia: a 20-year review. J Pediatr Surg. 1988;23:735–9. 3171843 10.1016/s0022-3468(88)80414-7

[11] Elhalaby EA Abo Sikeena MH. Delayed presentation of congenital diaphragmatic hernia. Pediatr Surg Int. 2002;18:480–5. 12415386 10.1007/s00383-002-0743-1

[12] Zedan M El-Ghazaly M Fouda A. Tension gastrothorax: a case report and review of literature. J Pediatr Surg. 2008;43:740–3. 18405725 10.1016/j.jpedsurg.2007.10.072

[13] Næss PA Wiborg J Kjellevold K. Tension gastrothorax: acute life-threatening manifestation of late onset congenital diaphragmatic hernia (CDH) in children. Scand J Trauma Resusc Emerg Med. 2015;23:49. 26104782 10.1186/s13049-015-0129-8 PMC4477604

[14] Pelech N Harikrishnan V Allagoa B. Tension gastrothorax - a life-threatening late presentation of congenital diaphragmatic hernia. Arch Dis Child. 2022;107:387. 34521635 10.1136/archdischild-2021-322583

[15] Song IH. Tension gastrothorax in late-onset congenital diaphragmatic hernia, a rare but life-threatening condition: a case report. Medicine (Baltim). 2021;100:e24815. 10.1097/MD.0000000000024815 PMC7899837 33607846

[16] García-Regalado JF Navarro-Rojas MM. Gastrotórax a tensión como causa de muerte por shock obstructivo: caso clínico [Tension gastrothorax as a cause of death by obstructive shock - case report] [In Spanish]. Rev Chil Pediatr. 2014;85:476–80. 25697322 10.4067/S0370-41062014000400011

[17] Fuller G Cacala S Oosthuizen G. Tension gastrothorax-colothorax secondary to traumatic diaphragmatic hernia. Pediatr Emerg Care. 2010;26:299–301. 20386417 10.1097/PEC.0b013e3181d6db22

[18] Hooker R Claudius I Truong A. Tension gastrothorax in a child presenting with abdominal pain. West J Emerg Med. 2012;13:117–8. 22461941 10.5811/westjem.2011.4.6763 PMC3298226

[19] Guo R Zhang L Zhang S. Case report: emergency treatment of late-presenting congenital diaphragmatic hernia with tension gastrothorax in three Chinese children. Front Pediatr. 2023;11:1115101. 36816375 10.3389/fped.2023.1115101 PMC9929136

[20] Rathinam S Margabanthu G Jothivel G. Tension gastrothorax causing cardiac arrest in a child. Interact Cardiovasc Thorac Surg. 2002;1:99–101. 17669971 10.1016/s1569-9293(02)00064-6

[21] Ahn S Kim W Sohn CH. Tension viscerothorax after blunt abdominal trauma: a case report and review of the literature. J Emerg Med. 2012;43:e451–3. 22244294 10.1016/j.jemermed.2011.05.084

[22] Sridhar AV Nichani S. Late presenting congenital diaphragmatic hernia. Emerg Med J. 2004;21:261–2. 14988374 10.1136/emj.2003.007435 PMC1726251

[23] Snyder HS Salo DF Kelly PH. Congenital diaphragmatic hernia presenting as massive gastrothorax. Ann Emerg Med. 1990;19:562–4. 2331102 10.1016/s0196-0644(05)82190-0

[24] Al Ghafri M Al Sidairi I Nayar M. Late presentation of congenital diaphragmatic hernia: a case report. Oman Med J. 2014;29:223–5. 10.5001/omj.2014.57 PMC4052392 24936275

